# Draft genome sequence of *Ovulinia azaleae*, a quarantine pathogen known for causing azalea petal blight

**DOI:** 10.1128/mra.00838-25

**Published:** 2026-01-15

**Authors:** Yunxin Zhang, Yunfang Chen, Sizhu Zheng, Xiaojun Yang, Zhenchuan Mao, Guohua Chen, Jian Ling

**Affiliations:** 1 State Key Laboratory of Vegetable Biobreeding, Institute of Vegetables and Flowers, Chinese Academy of Agricultural Sciences 12661, Beijing, China; 2 Comprehensive Technology Center of Suzhou Customs, Suzhou, China; 3 Animal, Plant and Food Inspection Center, Nanjing Customs, Nanjing, China; University of Strathclyde, Glasgow, United Kingdom

**Keywords:** *Ovulinia azaleae*, azalea petal blight, genome

## Abstract

We report the first draft genome sequence of *Ovulinia azaleae* (strain CBS 680.88). The long-read assembly resulted in a 56.83 Mb genome with an N50 of 3.05 Mb and 10,707 predicted protein-coding genes. The high-quality *Ovulinia azaleae* genome resource provides insights into the mechanisms of azalea petal blight.

## ANNOUNCEMENT

*Ovulinia azaleae* is a quarantine pathogen that exclusively infects azalea flowers (1, 2). Initial symptoms manifest as 1 mm diameter, water-soaked beige spots that progress to brown blotches, leading to flower wilting and premature abscission (3). This pathogen poses a significant threat to the rhododendron industry in China. To further analyze the pathogenic mechanism of *Ovulinia azaleae*, the whole genome sequence of *Ovulinia azaleae* was analyzed.

Strain CBS 680.88 was acquired from the Westerdijk Fungal Biodiversity Institute (CBS culture collection) as a pre-cultured and ready-to-use agar plate. For DNA extraction, fungal mycelia were grown in corn glucose broth at 25°C for 3 weeks, then harvested, washed, and freeze-dried. High-molecular weight genomic DNA was isolated using the CTAB method (4), followed by purification with the Monarch Genomic DNA Purification Kit (T3010L). Species identity was confirmed by sequencing the ITS region using primers ITS1/ITS4 (5). A BLASTn (v2.17.0+) search against the NCBI GenBank database (accessed 24 June 2025) identified *Ovulinia azaleae* (accession no. Z73797.1) as the top hit.

For multi-platform sequencing, both short-read and long-read libraries were constructed from the same genomic DNA extraction. An RNA-seq library was prepared using total RNA isolated from fungal mycelia. Quality control for the short-read and RNA-seq data was performed with fastp (v0.21.0) (6), which removed reads with mean base quality scores below Q20. For the long-read data, basecalling and adapter trimming were performed using Dorado (v0.9.0) with the "sup" model (dna_r10.4.1_e8.2_400bps_sup@v5.0.0). Reads with a mean Q-score below 10 were filtered out, yielding 15.04 Gb of high-quality long-read data. Detailed information regarding library preparation kits, sequencing platforms, quality control, and sequencing statistics are provided in Table 1.

**TABLE 1:** Sequencing data output statistics of *Ovulinia azaleae^a^*

Data type	Short-read data	RNA-seq data	Long-read data
Library prep kit	Hieff NGS OnePot Pro DNA Library Prep Kit V4 (Yeasen Biotechnology)	VAHTS Universal V6 RNA-seq Library Prep Kit for MGI (Vazyme Biotech)	SQK-LSK114 kit (Oxford Nanopore Technologies)^*b*^
Platform	DNBSEQ-T7	DNBSEQ-T7	PromethION platform using R10.4.1 flow cells
PE	150 bp	150 bp	–^*d*^
Raw reads	102,425,560	45,185,756	2,332,836
Raw bases	15.36 Gb	6.78 Gb	15.41 Gb
Fold coverage	189×	115×	271×
Clean reads	102,418,162	45,181,656	2,277,562
Clean bases	15.29 Gb	6.69 Gb	15.04 Gb
N50	–^*d*^	–^*d*^	12,497
Quality control	fastp (v0.21.0, -q 20)	fastp (v0.21.0, -q 20)	Sup^*c*^ (Q-score > 10)

^*a*^ Library preparation and sequencing were performed according to the manufacturer's instructions.

^*b*^ Without fragmentation and size selection.

^*c*^ Sup(dna_r10.4.1_e8.2_400bps_sup@v5.0.0).

^*d*^ –, not applicable.

**Fig 1:**
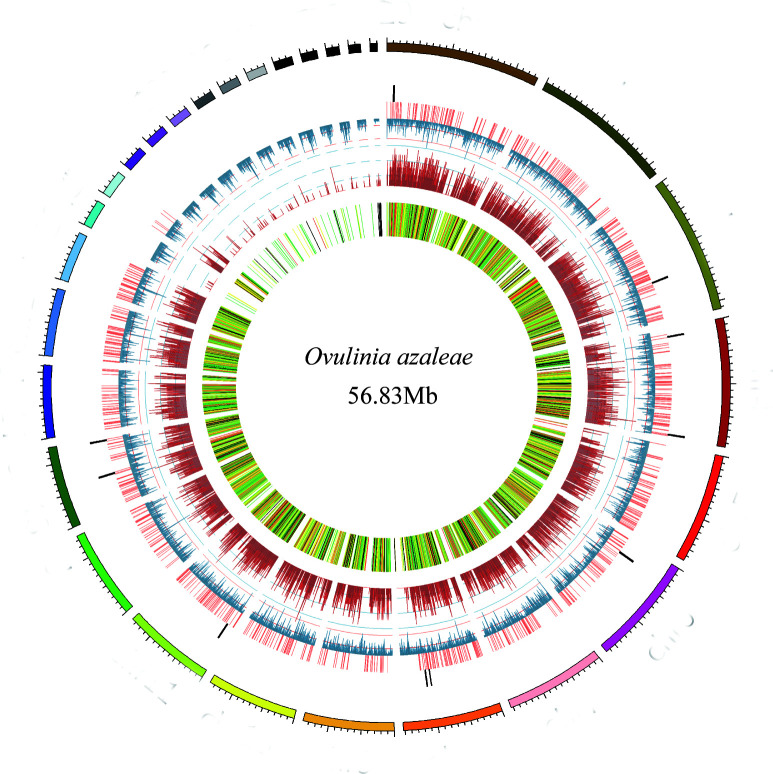
Genome features of *Ovulinia azaleae*. The outermost circle is the contigs. The bar charts from outside to inside in turn are secondary metabolite gene clusters (black), secreted proteins (orange), density of repetitive sequence (blue), gene density (dark red), and transcriptome (color heatmap).

Long reads were assembled independently using Canu (v2.2) (7) and NextDenovo (v2.5) (8), and the resulting assemblies were merged using QuickMerge (v0.3). The genome was polished via three rounds of Racon (v1.5.0) (9) with long reads, followed by three rounds of Pilon (v1.24) (10) with short reads. The final assembly genome was 56.83 Mb with 35.3% GC content, comprising 29 contigs with an N50 of 3.05 Mb. The largest contig spans 4.71 Mb (Fig. 1). For repetitive sequence annotation, a *de novo* library was constructed by RepeatModeler, and final repeats were masked by RepeatMasker (v4.0.9) (11), totaling 18.8 Mb (33.09% of the genome). The gene annotation for *Ovulinia azaleae* was generated by integrating *ab initio* and RNA-seq predictions. *Ab initio* predictions were completed through utilization of SNAP (v2.1.1) (12), AUGUSTUS (v3.3.2) (13), Gene Mark (v4.81), and Glimmer HMM (v3.0.4) (14). For transcriptome-based prediction, the transcripts were reconstructed with Spring Tie (v1.2.3) (15). These were integrated with EvidenceModeler (v1.1.1) (16), yielding 10,707 high-confidence genes. The assembly showed 97.2% completeness via BUSCO (v5.3.2) (17) against the fungi_odb10 dataset. In addition, functional annotation using NR, Swiss-Prot (2024_03), InterProScan (v88.0) (18), KOG, and Pfam (v35.0) (19) annotated 9,646 genes (90.09% of the total). Default parameters were used for all software unless otherwise specified.

## Data Availability

The genome and sequencing data have been deposited at NCBI under BioProject accession PRJNA1282300. The BioSample accession numbers for short-read sequencing, RNA-seq sequencing, and Nanopore sequencing are SAMN49645637, SAMN49645639, and SAMN49645638, respectively. The short-read sequencing reads are available in the NCBI Sequence Read Archive under accession number SRR34207007, the RNA-seq sequencing reads are accessible under accession number SRR34207008, and the Nanopore sequencing reads are accessible under accession number ERR15807457. This Whole Genome Shotgun project has been deposited in DDBJ/ENA/GenBank under accession number JBPJSN000000000. The version described in this paper is the first version, JBPJSN000000000. The genome annotation files generated in this study have been deposited in the Zenodo repository DOI: https://doi.org/10.5281/zenodo.17311068.
